# Antidepressant use in Sweden: an intersectional multilevel analysis of individual heterogeneity and discriminatory accuracy (MAIHDA)

**DOI:** 10.1177/1403494821993723

**Published:** 2021-02-23

**Authors:** Hanna Ljungman, Maria Wemrell, Kani Khalaf, Raquel Perez-Vicente, George Leckie, Juan Merlo

**Affiliations:** 1 Unit for Social Epidemiology, Lund University, Sweden; 2 Department of Gender Studies, Lund University, Sweden; 3 Center for Multilevel Modelling, University of Bristol, UK; 4 Center for Primary Health Care Research, Region Skåne, Sweden

**Keywords:** Antidepressants, socioeconomic factors, intersectionality, MAIHDA, Sweden

## Abstract

**Introduction:**

Antidepressants are among the most commonly prescribed drugs in Sweden. However, we lack detailed knowledge on the socioeconomic and demographic distribution of antidepressant use in the population. To fill this gap, we performed an intersectional multilevel analysis of individual heterogeneity and discriminatory accuracy.

**Methods:**

Analysing all Swedish residents older than 10 years (*n*=8,190,990), we measured the absolute risk of antidepressant use across 144 intersectional strata defined by combinations of age, gender, income, country of birth and psychiatric diagnosis. We calculated the strata-specific absolute risk of antidepressant use in a series of multilevel logistic regression models. By means of the variance partitioning coefficient and the area under the receiver operating characteristic curve, we quantified the discriminatory accuracy of the intersectional contexts (i.e. strata) for discerning those who use antidepressants from those who do not.

**Results:**

The absolute risk of antidepressant use ranged between 0.93% and 24.78% among those without a psychiatric diagnosis, and between 21.41% and 77.56% among those with a psychiatric diagnosis. Both the variance partitioning coefficient of 41.88% and the area under the receiver operating characteristic curve of 0.81 were considerable.

**Conclusions:**

Besides overt psychiatric diagnoses, our study shows that antidepressant use is mainly conditioned by age, which might express the embodiment of socioeconomic conditions across the individual life course. Our analysis provides a detailed and highly discriminatory mapping of the heterogeneous distribution of antidepressant use in the Swedish population, which may be useful in public health management.

## Introduction

Antidepressant medications are among the most commonly prescribed drugs in Europe and around the world [1, 2]. Sweden is one of the top five Organisation for Economic Co-operation and Development (OECD) countries with the highest prescription rates for antidepressants [1]. Between 2006 and 2011, the prevalence of antidepressant use in Sweden was relatively stable [2]. However, since then, antidepressant use has steadily increased. The rapid growth of antidepressant use worldwide has prompted discussion on why this increase is occurring, and whether antidepressants are appropriately prescribed.

While the existence of a psychiatric diagnosis is a main reason for using antidepressants, it is well established that low socioeconomic position (SEP) (e.g. low income, low educational achievement and unemployment) is associated with a higher risk of mental health disorders [3–6]. Although the direction of causality between SEP and mental health is debated (i.e. whether low SEP promotes the development of poor mental health or having poor mental health leads to low SEP), it is clear that a strong relationship exists. In addition, gender, marital status and country of birth all outline the life circumstances that promote or limit an individual’s exposure to stressors and the availability of social support and personal resources [3, 4] across the life course. These psychiatric risk factors are more common among lower SEP groups [4].

A few Scandinavian studies have demonstrated a higher prevalence of antidepressant prescriptions among women, people with low educational achievement and individuals who are unemployed, have a low income, or live alone [5, 7, 8]. In contrast, a Finnish study found that low SEP was associated with fewer antidepressant prescriptions among men, whereas no association was found among women. However, both men and women with low SEP had a higher prevalence of suicides [9], which suggests socioeconomic inequalities in access to treatment and mental health services.

In order to further our understanding of the sociodemographic distribution of antidepressant use in the population, intersectionality theory provides a suitable framework. Emerging from feminist theory and striving towards an understanding of how systems of oppression interact, an intersectional perspective directs attention to the ways in which social categories and systems that determine the distribution of resources and power create overlapping contexts of privilege and disadvantage or discrimination [10]. Intersectionality theory supports the construction of various intersectional strata defined by the combination of several socioeconomic and demographic dimensions that affect antidepressant use. Here, these factors are viewed as intersecting rather than separate contextual dimensions, which improves our understanding of the demographic and socioeconomic heterogeneity in the population.

To operationalise the above outlined intersectional perspective, we applied multilevel analysis of individual heterogeneity and discriminatory accuracy (MAIHDA) [11–13]. MAIHDA conceptualises the intersectional strata as social contexts rather than as individual characteristics [11]. It allows us to distinguish additive from interactive effects and provides information on the accuracy of the intersectional strata for discriminating individuals in the population that use antidepressants from those that do not. In turn, this information can be used for more precise public health policies following the framework of proportionate universalism [14].

Therefore, the aim of this study was to provide a better mapping of the demographic and socioeconomic distribution of antidepressant use in the Swedish population between 2006 and 2011. We aim to provide an improved basis for decision-making in future public health interventions that target disparities in access to mental healthcare treatment or the inappropriate use of antidepressants.

## Methods

### Data sources

We created a database linking the register of the total population (TPR) and the longitudinal integrated database for health insurance and labour market studies (LISA), administered by Statistics Sweden (SCB), as well as the National Patient Register (NPR), the Cause of Death Register, and the Swedish Prescribed Drug Register (SPDR) directed by the National Board of Health and Welfare (NBHW). The NPR records all discharge diagnoses from the hospital including outpatient specialised care according to the International Classification of Diseases and Causes of Death, 10th edition (ICD-10). The SPDR records information on all of the medications dispensed from Swedish pharmacies according to the anatomical therapeutic chemical (ATC) classification system. All registers were linked through the unique 12-digit personal registration number given to every Swedish resident. However, to ensure the confidentiality of personal information, SCB assigned arbitrary serial numbers to each personal number to anonymise the database prior to research use. The regional ethics review board in southern Sweden and the data safety committees from the NBHW and SCB approved the construction of the database.

### Study population

**Figure 1.**
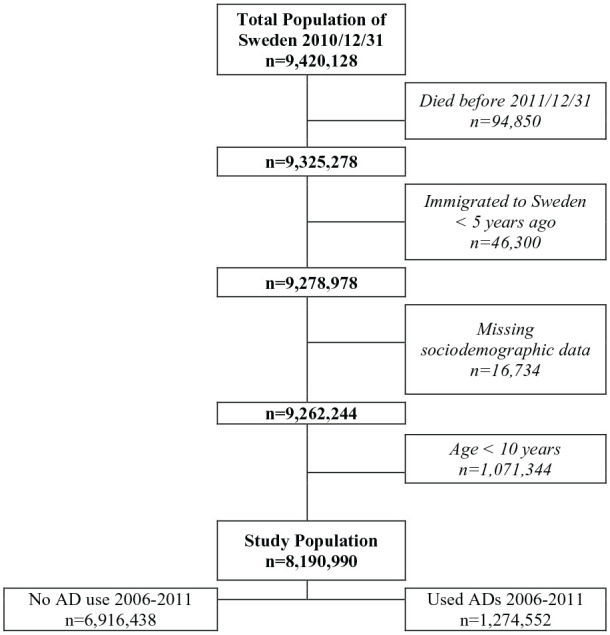
Flow chart documenting inclusion criteria, exclusion criteria and the total number of individuals included in the study population.

According to the TPR there were 9,420,128 individuals registered as residents in Sweden by 31 December 2010. From this population, 94,850 people had died before 31 December 2011 and were therefore excluded. In order to obtain reliable information on previous psychiatric diagnoses, we omitted 46,300 individuals who had lived in Sweden for less than 5 years before the baseline date. Thereafter, we excluded 16,734 individuals with missing variable information. We also excluded 1,071,344 children under the age of 10 years, as the prescription of antidepressants to those aged 9 years and younger has been rare in Sweden, 0.13–0.14 per 1000 children in 2006–2009 [15]. The final study population consisted of 8,190,990 Swedish residents over 10 years of age, with complete register information (Figure 1).

### Assessment of variables

The outcome variable was antidepressant use, defined by the dispensation of at least one antidepressant medication (ATC code: N06A) from a Swedish pharmacy between 2006 and 2011.

Gender was coded as a binary variable, either man or woman, based on the legal gender status of individuals. Age at the baseline date was categorised into six intervals: (a) 10–19 years; (b) 20–34 years; (c) 35–49 years; (d) 50–64 years; (e) 65–79 years; and (f) ⩾80 years. These age groups approximate life course periods which affect working life and socioeconomic position [16] (infancy, childhood and adolescence; early adulthood; early midlife; later midlife; retirement; and old age).

Country of birth was coded into a binary variable, in which individuals born in Sweden were labelled as natives and those who were born outside of Sweden were labelled as immigrants.

We created the income variable by using individualised cumulative household disposable income from 2000, 2005 and 2010. The total disposable income of a family was divided by the number of people in the family relative to different weights for adults and children, according to criteria from Statistics Sweden [17]. Using the total Swedish population, we computed 25 groups by quantiles in 2000, 2005 and 2010. Thereafter, we added the values from the 3 years, obtaining values with a range from 3 (lowest cumulative income) to 75 (highest cumulative income). Finally, we categorised this cumulative income into three categories (low, middle and high) by dividing the range into tertiles. Individuals with missing values on income during 2000 or 2005, including those who had immigrated to Sweden prior to 2005, were assigned the tertile values based only on their income during 2010. No individuals in the study population had missing income data for 2010.

A diagnosed psychiatric disorder was defined by the ICD-10 codes F01–F99 from a hospital or outpatient specialised care, not including diagnoses made at the primary care level. Psychiatric diagnosis is a strong determinant of antidepressant use and therefore a relevant variable to consider in our analysis. Rather than using it as a control variable we included it as a dimension in the intersectional strata, because mental ill-health can contribute towards social stratification not least through affecting access to societal services including appropriate healthcare [18]. However, the intersectional analysis of those with and without a psychiatric diagnosis might also be considered separately.

We generated 144 intersectional strata by combining the six categories of age, two categories of gender, three categories of income, two categories of country of birth and two categories of psychiatric diagnosis.

### Statistical analysis

We performed an intersectional MAIHDA [12, 13, 19] with individuals (level 1) clustered within intersectional strata (level 2). We obtained the absolute risk (AR) of using antidepressants by multilevel logistic regression models with back transformation of predicted log odds in each stratum to the probability scale. We estimated the AR with 95% credible intervals (CIs) associated with each stratum. These predictions are so-called ‘shrinkage’ estimates (based on predicted random effects).

Model 1 was a simple intersectional model that only included an intercept and a random effect for the intersectional strata with no covariates. We calculated the variance partition coefficient (VPC), which indicates the share of the total individual variance in the latent propensity of antidepressant use that resides at the intersectional strata level [20].

We also calculated the area under the receiver operator characteristic curve (AUC) [21, 22] by using the predicted probabilities. The AUC measures the accuracy of the information provided by the variables in the model for discriminating individuals in the population that use antidepressants from those that do not. The value of the AUC can range from 0.5, indicating the absence of discriminatory accuracy (DA), to 1, representing perfect discrimination. We use the classification provided by Hosmer and Lemeshow to define the DA as: (a) ‘absent or very low’ (AUC 0.5–0.6); (b) ‘poor’ (>0.6–0.7); (c) ‘acceptable’ (>0.7–0.8); (d) ‘excellent’ (>0.8–0.9); or (e) ‘outstanding’ (>0.9–1) [23].

Finally, model 1 was used to map the distribution of antidepressant use in the Swedish population based on the predicted strata-specific risks of antidepressant use from the predicted strata random effects (i.e. shrunken residuals).

Model 2 represents the partially adjusted model in which we expanded model 1 by adding one covariate at a time: model 2a (age), 2b (gender), 2c (income), 2d (country of birth) and 2e (psychiatric diagnosis). By calculating the proportional change in the between-strata variance (PCV) in relation to model 1, we were able to quantify the proportion of between-strata variance explained by each of the variables making up the intersectional strata.

We also calculated the AUC based on the prediction from the fixed effects of the partially adjusted models. While the model 1 prediction is based only on the intercept and stratum random effects, the model 2 prediction is decomposed into fixed and random effect components. In the different model 2s we aimed to understand the AUC based on the fixed effect of each variable. Thus, the AUC in model 2 measures the accuracy of the information provided by one specific variable for discriminating individuals in the population that use antidepressants from those that do not.

Model 3 represents the intersectional interaction model. It expands on models 1 and 2 by simultaneously including all the variables used to construct the intersectional strata as covariates with fixed effect regression coefficients. In the absence of strata-specific statistical interactions, the main effects of the variables used to construct the strata would completely explain the between-strata variance and all 144 strata random effects would equal zero. However, if this is not the case, and if no relevant variables were omitted, the strata random effects represent the existence of statistical interaction effects between the variables. In this way, model 3 differentiates the main (additive) effects from the interaction effects. Thus, a possible strata variance would illustrate the existence of intersectional multiplicative interaction of effects in relation to the variables included in the model.

We calculated the interaction with 95% CIs on the probability scale for each intersectional stratum by subtracting the risk of antidepressant use based on the main effects only from the total risk of antidepressant use (based on main and interaction effects). A positive probability difference means that individuals in that intersectional strata have a higher risk than expected based on the simple addition of risks conveyed by the variables that define the intersectional strata, whereas a negative proportion represents a lower risk than expected.

Statistical analyses were run using MLwiN 3.00 by calling it from within Stata 14.1 using the *runmlwin* [24] command. The estimations were performed using Markov chain Monte Carlo (MCMC) methods. The Stata do-file (script) used in the analyses is provided in the Supplemental material.

## Results

A total of 1,274,552 individuals purchased at least one antidepressant medication from a pharmacy between 2006 and 2011, representing an overall period prevalence of 15.56% among the 8,190,990 Swedish residents over the age of 10 years. The socioeconomic and demographic profile of the sample population is summarised in Table I.

**Table I.** Descriptive statistics for socioeconomic and demographic differences of antidepressant use in the Swedish population, as well as the prevalence of antidepressant use for each variable.

Variable		*n*	Purchase of an antidepressant		
			No	Yes	%
**Study population**		8,190,990	6,916,438	1,274,552	15.56
**Age**	10–19	1,118,533	1,065,270	53,263	4.76
	20–34	1,765,779	1,550,856	244,923	13.64
	35–49	1,927,739	1,581,037	346,702	17.98
	50–64	1,751,270	1,414,224	337,046	19.25
	65–79	1,184,678	986,156	198,522	16.76
	⩾80	412,991	318,895	94,096	27.78
**Gender**	Women	4,127,639	3,305,227	822,412	19.92
	Men	4,063,351	3,611,211	452,140	11.13
**Income**	Low	2,643,475	2,213,726	429,749	16.26
	Middle	2,812,857	2,350,998	461,859	16.42
	High	2,734,658	2,351,714	382,944	14.00
**Country of birth**	Native^a^	6,906,663	5,837,216	1,069,447	15.48
	Immigrant	1,284,327	1,079,222	205,105	15.97
**Psychiatric diagnosis**	No	7,528,977	6,656,723	871,354	11.57
	Yes	662,913	259,715	403,198	60.82

*n*; number of individuals.

%: prevalence of antidepressant use for each variable.

a Born in Sweden.

**Figure 2.**
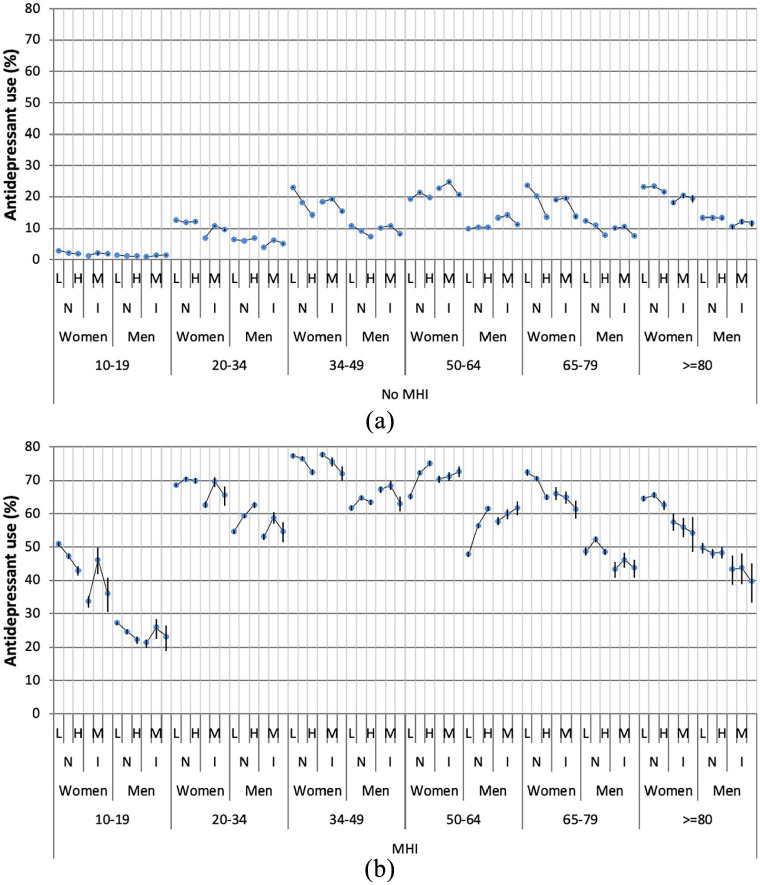
(a) Prevalence of antidepressant use (model 1) in individuals without a psychiatric diagnosis by intersectional strata defined by age, gender, country of birth (N for natives, and I for immigrants) and low (L), middle (M) and high (H) income levels. The association between the three levels of income and antidepressant use is illustrated by circles connected by thin lines and crossed by vertical lines representing 95% credible intervals (CIs). (b) Prevalence of antidepressant use (model 1) in individuals with a psychiatric diagnosis by intersectional strata defined by age, gender, country of birth (N for natives, and I for immigrants) and low (L), middle (M), and high (H) income levels. The association between the three levels of income and antidepressant use is illustrated by circles connected by thin lines and crossed by vertical lines representing 95% CIs.

Figure 2 maps the simple intersectional (model 1) strata-specific ARs for antidepressant use, which correspond with the prevalence values. For those without a psychiatric diagnosis (Figure 2(a)) we found that the lowest AR for antidepressant use was among 10–19-year-old low-income immigrant men (AR 0.93%) and highest among middle-income immigrant women aged 50–64 years (AR 24.78%). Conversely, in the strata with a psychiatric diagnosis (Figure 2(b)), the AR ranged from 21.41% among 10–19-year-old low-income immigrant men to 77.56% among 35–49-year-old low-income immigrant women. Furthermore, antidepressant use is shown to increase with rising age among the strata without a psychiatric diagnosis. However, among those with a psychiatric diagnosis, the prevalence of antidepressant use increases rapidly until age 35–49 years then decreases gently.

Table II shows the AUC, between-strata variance, VPC and PCV with 95% CIs for the three models, as well as the odds ratio for the variables. The VPC from model 1 indicates that as much as 41.88% of the total variance among individuals resides at the intersectional strata level. We found that having a psychiatric diagnosis (model 2e) explained a large amount (PCV 71.31%) of the between-strata variance. Age (model 2a) also accounted for a considerable amount of the variance (PCV 18.57%), whereas the other intersectional dimensions explained little to none of the between-strata variance. The intersectional interaction model 3 reduced the between-strata variance considerably (PCV 95.36%) indicating that the differences between strata were mainly due to additive rather than interaction effects of the variables (4.64%) used to define the strata. The VPC pertaining to model 3 was only 3.27%.

The AUC of the simple intersectional model based on the random effects was 0.81, suggesting an acceptable DA. The age-specific AUC (model 2a) was 0.58 and the psychiatric diagnosis-specific AUC (model 2e) was 0.75, whereas the AUCs for the other variables were close to 0.5 (i.e. absence of DA) suggesting that gender, income and country of birth alone are poor predictors of antidepressant use. The AUC of model 3, based on the fixed effects of all the variables defining the intersectional strata, was the same as that of model 1, based on the random effects only (AUC 0.81). This again indicates that the interaction of effects in model 3 (which is captured as random effects) has effectively no relevance. Because the intersectional interaction effects were small overall, we did not investigate specific strata interactions.

**Table II.** Fixed effects of antidepressant use: models 1–3.

**Exposure**		**Model 1:** empty model	**Model 2a:** age adjusted	**Model 2b:** gender adjusted	**Model 2c:** income adjusted	**Model 2d:** country of birth adjusted	**Model 2e:** psychiatric diagnosis adjusted	**Model 3:** interaction effects
**Age**	10–19		0.15 (0.11–0.24)					0.15 (0.13–0.18)
	20–34		0.47 (0.39–0.58)					0.60 (0.53–0.71)
	35–49		Reference					Reference
	50–64		1.04(0.87–1.21)					1.04 (0.90–1.17)
	65–79		0.64 (0.56–0.70)					0.78 (0.70–0.87)
	⩾ 80		0.93 (0.54–1.45)					0.80 (0.69–0.96)
**Gender**	Male			Reference				Reference
	Female			1.63 (1.22–1.91)				1.92 (1.81–2.06)
**Income**	Low				1.24 (0.89–1.46)			1.05 (0.95–1.19)
	Middle				1.10 (0.97–1.27)			1.14 (1.02–1.26)
	High				Reference			Reference
**Country of birth**	Native					Reference		Reference
	Immigrant					0.67 (0.55–0.88)		0.92 (0.83–1.02)
**Psychiatric Diagnosis**	No						Reference	Reference
	Yes						10.97 (9.34–12.95)	12.82 (11.77–14.09)
**AUC**		0.81 (0.81–0.81)	0.58* (0.58–0.58)	0.56* (0.56–0.56)	0.50* (0.50–0.50)	0.51* (0.51–0.51)	0.75* (0.75–0.75)	0.81* (0.81–0.81)
**Variance**		2.37 (1.88–2.98)	1.93 (1.53–2.45)	2.31 (1.83–2.91)	2.38 (1.90–2.99)	2.42 (1.91–3.04)	0.68 (0.54–0.86)	0.11 (0.09–0.14)
**VPC (%)**		41.88%	37.00%	41.25%	41.96%	42.39%	17.14%	3.27%
**PCV (%)**			18.57%	2.53%	–0.42%	–2.11%	71.31%	95.36%

Odds ratios, area under the receiver operating curve (AUC), between-intersectional strata variance, variance partition coefficient (VPC) and proportional change in variance (PCV) of antidepressant use with 95% credible intervals (CIs) for the simple intersectional model 1 (simple components of variance analysis), the partially adjusted model 2 and the intersectional interaction model 3 (adjusted for all variables) are shown in this table.

* The AUCs of models 2 and 3 are obtained from the fixed effects only.

## Discussion

By applying an intersectional perspective, our study provides a better mapping of the socioeconomic and demographic distribution of antidepressant use in the Swedish population. We found that for those without a psychiatric diagnosis, the propensity of being dispensed an antidepressant from a pharmacy in Sweden was highest among middle-income immigrant women aged 50–64 years, whereas for those with a psychiatric diagnosis, it was highest among low-income immigrant women aged 35–49 years. For those with and without a psychiatric diagnosis, young immigrant men with medium or low income, respectively, used antidepressants the least. Furthermore, 41.88% of the total variance among individuals was located at the intersectional strata level, indicating that the intersectional context conditioned individual use of antidepressants.

We found that antidepressant use increased with age in men and women without a psychiatric diagnosis. Moreover, the age variable accounted for much more of the between-strata variance than the other variables. However, this needs to be properly interpreted. It may not mean that the variables of gender, income and country of birth are irrelevant, rather, the age gradient might be a result of the embodiment of socioeconomic and demographic factors across the life course. That is, the negative consequences of low income, gender and immigration may accumulate over the life course and lead to a higher risk of antidepressant use later in life [25, 26].

While we cannot determine whether antidepressants are being over or under-prescribed from our study, we can speculate about potential disparities in access to treatment based on previous literature. Women are more likely to seek out mental healthcare, therefore increasing the likelihood that their depression is detected and treated [27], whereas depression in men typically presents itself in different ways other than the classic depressive symptoms (i.e. through addiction, aggression, etc.), thus men’s mental health problems are often not recognised as such and are therefore under-treated [28]. Furthermore, the slightly lower risk of antidepressant use among immigrants found in this study may be an indication of barriers to accessing healthcare, rather than a better mental health status. Previous studies have found that despite having a higher risk of mental health problems [29], immigrants utilise healthcare to a lesser extent than natives in Sweden because of long waiting times, language difficulties and discrimination [30].

Our findings provide motivation for precision public health interventions following Michael Marmot’s idea of proportionate universalism, meaning that policies and interventions should be universal, not targeted, but with a ‘scale and intensity proportionate to the level of disadvantage’ [14]. Our intersectional MAIHDA methodology operationalises this idea by providing information on the DA of the contexts that define the intersectional strata. Since the analysis yielded a considerably high DA (i.e. VPC 41.88%, AUC 0.81), our study suggests that public health interventions, such as targeting disparities in access to treatment or the inappropriate use of antidepressants, being always universal, could be focused on intersectional strata with a very high use of antidepressants.

As noted, our analysis revealed very small statistical interaction effects. It should be noted here that in certain contrast with intersectionality theory, in which interaction between co-existing dimensions of social stratification is referred to in broader terms and not necessarily quantified, quantitative measures of the health effects of occupying certain intersectional strata can be decomposed into additive and interaction effects. As discussed elsewhere [12], we argue that intersectional heterogeneities, mirroring the societal distribution of resources that condition health, are relevant irrespective of whether the effects are due to underlying additive or interactive mechanisms.

This study was based on the analysis of the total Swedish population, which allowed for a representative study population and reliable dispensing data. Examining the prevalence over a 6-year period allowed us to capture all types of antidepressant users including new users, continued users and discontinued users during the study period. However, the findings from this study should be interpreted in the context of its limitations. The SPDR only provides information on dispensed prescriptions, which may not reflect the actual use of those medications. In addition, the psychiatric diagnosis variable only captures the individuals with severe mental health problems, and not those who are diagnosed with a milder form of a psychiatric disorder by a primary care doctor. Furthermore, this study was based on individuals residing in Sweden, a country with a strong social welfare system with universal healthcare, therefore the generalisability of the results to other healthcare systems may be limited.

In conclusion, through simultaneously examining several and interrelated socioeconomic and demographic factors, this study provides a better mapping of the distribution of antidepressant use in the Swedish population. Our findings provide relevant information regarding the prevalence of antidepressant use in Sweden in relation to socioeconomic and demographic factors that should be taken into consideration when developing public health interventions.

## Supplemental Material

sj-pdf-1-sjp-10.1177_1403494821993723 – Supplemental material for Antidepressant use in Sweden: an intersectional multilevel analysis of individual heterogeneity and discriminatory accuracy (MAIHDA) Click here for additional data file. Supplemental material, sj-pdf-1-sjp-10.1177_1403494821993723 for Antidepressant use in Sweden: an intersectional multilevel analysis of individual heterogeneity and discriminatory accuracy (MAIHDA) by Hanna Ljungman, Maria Wemrell, Kani Khalaf, Raquel Perez-Vicente, George Leckie and Juan Merlo in Scandinavian Journal of Public Health
