# Supplementary material for: Antidepressant use in Sweden: an intersectional multilevel analysis of individual heterogeneity and discriminatory accuracy (MAIHDA)
Source: Scand J Public Health. 2021 Feb 23;50(3):395–403. doi: 10.1177/1403494821993723 (PMC9096592; doi:10.1177/1403494821993723)
Supplement: sj-pdf-1-sjp-10.1177_1403494821993723 – Supplemental material for Antidepressant use in Sweden: an intersectional multilevel analysis of individual heterogeneity and discriminatory accuracy (MAIHDA) [file sj-pdf-1-sjp-10.1177_1403494821993723.pdf]

```
global MLwiN_path "C:\Program Files\MLwiN v3.02\mlwin.exe"



********************
 ********************
*MODEL 1:Null model
 ********************
 ********************

use final_ml, clear
* IGLS estimation, for MCMC initial values
runmlwin prop cons , ///
  level2(gr: cons) ///
  level1(gr:) ///
  discrete(distribution(binomial) link(logit) denom(denom) mql1) ///
  nopause

* MCMC
runmlwin prop cons , ///
  level2(gr: cons, residuals(u,savechains("u.dta",replace))) ///
  level1(gr:) ///
  discrete(distribution(binomial) link(logit) denom(denom)) ///
  mcmc( burnin(10000) chain(50000) thin(10)  savechains("b.dta", replace)) ///
  initsprevious ///
  nopause


scalar m1sigma2u = [RP2]var(cons)
*scalar m1sigma2e = _pi^2/3
display "VPC_u = " %9.4f m1sigma2u/(m1sigma2u + 3.29)
compress
save "m1.dta", replace


*-------------------------------------------------------------------------------*
* PREPARE FIXED-PART PAREMETER CHAINS
*-------------------------------------------------------------------------------*

use "b.dta", clear
drop deviance RP2_var_cons_ OD_bcons_1
rename FP1_* b_*
format %9.2f b_*
compress
save "b_prepped.dta", replace
isid iteration
codebook iteration, compact

*-------------------------------------------------------------------------------*
* PREPARE inter RANDOM EFFECTS CHAINS
*-------------------------------------------------------------------------------*


use "u.dta", clear
drop residual idnum
rename value u
format %9.2f u
sort gr iteration
order gr iteration
```

```
compress
save "u_prepped.dta", replace
isid gr iteration
codebook iteration, compact



*-------------------------------------------------------------------------------*
* MERGE DATA, FIXED-PART PARAMETER AND RANDOM EFFECT CHAINS TOGETHER
*-------------------------------------------------------------------------------*

use "final_ml", clear

cross using "b_prepped.dta"
merge m:1 gr iteration using "u_prepped.dta", nogenerate assert(match)
save "data_prepped1.dta", replace

use "data_prepped1.dta", clear
gen pk=invlogit(b_cons*cons+u)

gcollapse (p2.5) pk_lo = pk (mean) pk_mn = pk (p97.5) pk_hi = pk, by(gr)
order pk_mn
sort pk_mn

gen pk_rank=_n

save model1, replace



 ********************
 ********************
 *MODEL 2 : Age
 ********************
 ********************


use final_ml, clear
* IGLS estimation, for MCMC initial values
runmlwin prop cons age1-age2 age4-age6, ///
  level2(gr: cons) ///
  level1(gr:) ///
  discrete(distribution(binomial) link(logit) denom(denom) mql1) ///
  nopause

* MCMC
runmlwin prop cons age1-age2 age4-age6, ///
  level2(gr: cons, residuals(u,savechains("u.dta",replace))) ///
  level1(gr:) ///
  discrete(distribution(binomial) link(logit) denom(denom)) ///
  mcmc( burnin(10000) chain(50000) thin(10)  savechains("b.dta", replace)) ///
  initsprevious ///
  nopause

runmlwin, or

 scalar m1sigma2u = [RP2]var(cons)
*scalar m1sigma2e = _pi^2/3
display "VPC_u = " %9.4f m1sigma2u/(m1sigma2u + 3.29)
```

```
compress
save "m2.dta", replace







*--------------------------------------------------------------------------------*
* PREPARE FIXED-PART PAREMETER CHAINS
*--------------------------------------------------------------------------------*

use "b.dta", clear
drop deviance RP2_var_cons_ OD_bcons_1
rename FP1_* b_*
format %9.2f b_*
compress
save "b_prepped.dta", replace
isid iteration
codebook iteration, compact

*--------------------------------------------------------------------------------*
* PREPARE inter RANDOM EFFECTS CHAINS
*--------------------------------------------------------------------------------*


use "u.dta", clear
drop residual idnum
rename value u
format %9.2f u
sort gr iteration
order gr iteration
compress
save "u_prepped.dta", replace
isid gr iteration
codebook iteration, compact



*--------------------------------------------------------------------------------*
* MERGE DATA, FIXED-PART PARAMETER AND RANDOM EFFECT CHAINS TOGETHER
*--------------------------------------------------------------------------------*

use "final_ml", clear

cross using "b_prepped.dta"
merge m:1 gr iteration using "u_prepped.dta", nogenerate assert(match)
save "data_prepped2.dta", replace






 ********************
```

```
 ********************
 *MODEL 3 : Gender
 ********************
 ********************

 use final_ml, clear

 gen female=1 if male==0
 replace female=0 if fema==.
 save, replace
use final_ml, clear


* IGLS estimation, for MCMC initial values
runmlwin prop cons female, ///
  level2(gr: cons) ///
  level1(gr:) ///
  discrete(distribution(binomial) link(logit) denom(denom) mql1) ///
  nopause

* MCMC
runmlwin prop cons female, ///
  level2(gr: cons, residuals(u,savechains("u.dta",replace))) ///
  level1(gr:) ///
  discrete(distribution(binomial) link(logit) denom(denom)) ///
  mcmc( burnin(10000) chain(50000) thin(10)  savechains("b.dta", replace)) ///
  initsprevious ///
  nopause

runmlwin, or

 scalar m1sigma2u = [RP2]var(cons)
*scalar m1sigma2e = _pi^2/3
display "VPC_u = " %9.4f m1sigma2u/(m1sigma2u + 3.29)
compress
save "m3.dta", replace







*-------------------------------------------------------------------------------*
* PREPARE FIXED-PART PAREMETER CHAINS
*-------------------------------------------------------------------------------*

use "b.dta", clear
drop deviance RP2_var_cons_ OD_bcons_1
rename FP1_* b_*
format %9.2f b_*
compress
save "b_prepped.dta", replace
isid iteration
codebook iteration, compact

*-------------------------------------------------------------------------------*
* PREPARE inter RANDOM EFFECTS CHAINS
```

```
*--------------------------------------------------------------------------------*


use "u.dta", clear
drop residual idnum
rename value u
format %9.2f u
sort gr iteration
order gr iteration
compress
save "u_prepped.dta", replace
isid gr iteration
codebook iteration, compact



*--------------------------------------------------------------------------------*
* MERGE DATA, FIXED-PART PARAMETER AND RANDOM EFFECT CHAINS TOGETHER
*--------------------------------------------------------------------------------*

use "final_ml", clear

cross using "b_prepped.dta"
merge m:1 gr iteration using "u_prepped.dta", nogenerate assert(match)
save "data_prepped3.dta", replace




  ********************
 ********************
 *MODEL 4 : income
 ********************
 ********************



use final_ml, clear
* IGLS estimation, for MCMC initial values
runmlwin prop cons  income1 income2 , ///
  level2(gr: cons) ///
  level1(gr:) ///
  discrete(distribution(binomial) link(logit) denom(denom) mql1) ///
  nopause

* MCMC
runmlwin prop cons  income1 income2 , ///
  level2(gr: cons, residuals(u,savechains("u.dta",replace))) ///
  level1(gr:) ///
  discrete(distribution(binomial) link(logit) denom(denom)) ///
  mcmc( burnin(10000) chain(50000) thin(10)  savechains("b.dta", replace)) ///
  initsprevious ///
  nopause

runmlwin, or

 scalar m1sigma2u = [RP2]var(cons)
*scalar m1sigma2e = _pi^2/3
display "VPC_u = " %9.4f m1sigma2u/(m1sigma2u + 3.29)
compress
```

```
save "m4.dta", replace







*-------------------------------------------------------------------------------*
* PREPARE FIXED-PART PAREMETER CHAINS
*-------------------------------------------------------------------------------*

use "b.dta", clear
drop deviance RP2_var_cons_ OD_bcons_1
rename FP1_* b_*
format %9.2f b_*
compress
save "b_prepped.dta", replace
isid iteration
codebook iteration, compact

*-------------------------------------------------------------------------------*
* PREPARE inter RANDOM EFFECTS CHAINS
*-------------------------------------------------------------------------------*


use "u.dta", clear
drop residual idnum
rename value u
format %9.2f u
sort gr iteration
order gr iteration
compress
save "u_prepped.dta", replace
isid gr iteration
codebook iteration, compact



*-------------------------------------------------------------------------------*
* MERGE DATA, FIXED-PART PARAMETER AND RANDOM EFFECT CHAINS TOGETHER
*-------------------------------------------------------------------------------*

use "final_ml", clear

cross using "b_prepped.dta"
merge m:1 gr iteration using "u_prepped.dta", nogenerate assert(match)
save "data_prepped4.dta", replace





   ********************
 ********************
 *MODEL 5 : Immigration status
 ********************
```

```
 ********************

use final_ml, clear
* IGLS estimation, for MCMC initial values
runmlwin prop cons  imm , ///
  level2(gr: cons) ///
  level1(gr:) ///
  discrete(distribution(binomial) link(logit) denom(denom) mql1) ///
  nopause

* MCMC
runmlwin prop cons  imm, ///
  level2(gr: cons, residuals(u,savechains("u.dta",replace))) ///
  level1(gr:) ///
  discrete(distribution(binomial) link(logit) denom(denom)) ///
  mcmc( burnin(10000) chain(50000) thin(10)  savechains("b.dta", replace)) ///
  initsprevious ///
  nopause

runmlwin, or

 scalar m1sigma2u = [RP2]var(cons)
*scalar m1sigma2e = _pi^2/3
display "VPC_u = " %9.4f m1sigma2u/(m1sigma2u + 3.29)
compress
save "m5.dta", replace


*-------------------------------------------------------------------------------*
* PREPARE FIXED-PART PAREMETER CHAINS
*-------------------------------------------------------------------------------*

use "b.dta", clear
drop deviance RP2_var_cons_ OD_bcons_1
rename FP1_* b_*
format %9.2f b_*
compress
save "b_prepped.dta", replace
isid iteration
codebook iteration, compact

*-------------------------------------------------------------------------------*
* PREPARE inter RANDOM EFFECTS CHAINS
*-------------------------------------------------------------------------------*


use "u.dta", clear
drop residual idnum
rename value u
format %9.2f u
sort gr iteration
order gr iteration
```

```
compress
save "u_prepped.dta", replace
isid gr iteration
codebook iteration, compact



*--------------------------------------------------------------------------------*
* MERGE DATA, FIXED-PART PARAMETER AND RANDOM EFFECT CHAINS TOGETHER
*--------------------------------------------------------------------------------*

use "final_ml", clear

cross using "b_prepped.dta"
merge m:1 gr iteration using "u_prepped.dta", nogenerate assert(match)
save "data_prepped5.dta", replace





   ********************
 ********************
 *MODEL 6 : MHI
 ********************
 ********************



use final_ml, clear
* IGLS estimation, for MCMC initial values
runmlwin prop cons  mental , ///
  level2(gr: cons) ///
  level1(gr:) ///
  discrete(distribution(binomial) link(logit) denom(denom) mql1) ///
  nopause

* MCMC
runmlwin prop cons  mental, ///
  level2(gr: cons, residuals(u,savechains("u.dta",replace))) ///
  level1(gr:) ///
  discrete(distribution(binomial) link(logit) denom(denom)) ///
  mcmc( burnin(10000) chain(50000) thin(10)  savechains("b.dta", replace)) ///
  initsprevious ///
  nopause

runmlwin, or

 scalar m1sigma2u = [RP2]var(cons)
*scalar m1sigma2e = _pi^2/3
display "VPC_u = " %9.4f m1sigma2u/(m1sigma2u + 3.29)
compress
save "m6.dta", replace
```

```
*------------------------------------------------------------------------------*
* PREPARE FIXED-PART PAREMETER CHAINS
*------------------------------------------------------------------------------*

use "b.dta", clear
drop deviance RP2_var_cons_ OD_bcons_1
rename FP1_* b_*
format %9.2f b_*
compress
save "b_prepped.dta", replace
isid iteration
codebook iteration, compact

*------------------------------------------------------------------------------*
* PREPARE inter RANDOM EFFECTS CHAINS
*------------------------------------------------------------------------------*


use "u.dta", clear
drop residual idnum
rename value u
format %9.2f u
sort gr iteration
order gr iteration
compress
save "u_prepped.dta", replace
isid gr iteration
codebook iteration, compact



*------------------------------------------------------------------------------*
* MERGE DATA, FIXED-PART PARAMETER AND RANDOM EFFECT CHAINS TOGETHER
*------------------------------------------------------------------------------*

use "final_ml", clear

cross using "b_prepped.dta"
merge m:1 gr iteration using "u_prepped.dta", nogenerate assert(match)
save "data_prepped6.dta", replace







 ********************
 ********************
 *MODEL 7 :Intersectional model
 ********************
 ********************


use final_ml, clear

* IGLS estimation, for MCMC initial values
```

```
runmlwin prop cons age1-age2 age4-age6 female income1 income2 imm mental  , ///
  level2(gr: cons) ///
  level1(gr:) ///
  discrete(distribution(binomial) link(logit) denom(denom) mql1) ///
  nopause

* MCMC
runmlwin prop cons age1-age2 age4-age6 female income1 income2 imm mental  , ///
  level2(gr: cons, residuals(u,savechains("u.dta",replace))) ///
  level1(gr:) ///
  discrete(distribution(binomial) link(logit) denom(denom)) ///
  mcmc( burnin(10000) chain(50000) thin(10)  savechains("b.dta", replace)) ///
  initsprevious ///
  nopause

runmlwin, or

 scalar m1sigma2u = [RP2]var(cons)
*scalar m1sigma2e = _pi^2/3
display "VPC_u = " %9.4f m1sigma2u/(m1sigma2u + 3.29)
compress
save "m7.dta", replace







*-------------------------------------------------------------------------------*
* PREPARE FIXED-PART PAREMETER CHAINS
*-------------------------------------------------------------------------------*

use "b.dta", clear
drop deviance RP2_var_cons_ OD_bcons_1
rename FP1_* b_*
format %9.2f b_*
compress
save "b_prepped.dta", replace
isid iteration
codebook iteration, compact

*-------------------------------------------------------------------------------*
* PREPARE inter RANDOM EFFECTS CHAINS
*-------------------------------------------------------------------------------*


use "u.dta", clear
drop residual idnum
rename value u
format %9.2f u
sort gr iteration
order gr iteration
compress
save "u_prepped.dta", replace
isid gr iteration
codebook iteration, compact
```

```
*-------------------------------------------------------------------------------*
* MERGE DATA, FIXED-PART PARAMETER AND RANDOM EFFECT CHAINS TOGETHER
*-------------------------------------------------------------------------------*

use "final_ml", clear

cross using "b_prepped.dta"
merge m:1 gr iteration using "u_prepped.dta", nogenerate assert(match)
save "data_prepped7.dta", replace

use "data_prepped7.dta", clear
gen pk=invlogit(b_cons*cons+u)

gcollapse (p2.5) pk_lo = pk (mean) pk_mn = pk (p97.5) pk_hi = pk, by(gr)
order pk_mn
sort pk_mn

gen pk_rank=_n

save model7, replace
```
